# Mapping affective pathways to compulsion: Insights from an aversive devaluation approach

**DOI:** 10.1556/2006.2025.00089

**Published:** 2025-11-19

**Authors:** Samantha N. Sallie, Violeta Casero, Saurabh Sonkusare, Valerie Voon

**Affiliations:** 1 Department of Psychiatry, University of Cambridge, Cambridge, United Kingdom; 2 Institute of Science and Technology for Brain-Inspired Intelligence, Fudan University, Shanghai, China

**Keywords:** addiction, avoidance, compulsivity, habit, negative emotionality, transdiagnostic

## Abstract

**Background and aims:**

Dysregulation in instrumental control systems is implicated in compulsivity, a transdiagnostic construct proposed to underlie diverse maladaptive behaviors. While habit formation in reward-based learning is well-characterized, its role in avoidance learning remains less understood. Habitual avoidance may contribute to compulsive symptoms by impairing emotion regulation, a well-established correlate of compulsivity. To define these mechanisms, this study examined negative emotionality as a pathway linking habitual avoidance to compulsive behaviors.

**Methods:**

Five hundred adults completed the Avoidance Dynamics Task (ADT), a novel online-administered aversive devaluation paradigm assessing avoidance learning and habit strength, alongside validated self-report measures of compulsive behaviors (alcohol use, binge eating, binge watching, gambling, obsessive-compulsive symptoms) and internalizing symptoms (depression, anxiety). Mediation analysis tested whether internalizing symptoms accounted for associations between habitual avoidance and compulsive behavior severity.

**Results:**

Habitual avoidance, indexed by perseverative responses to devalued threat versus control cues (*t* = 3.5, *p* = .002), showed small-to-moderate positive associations with avoidance urges (*ρ* = .28, *p* < .001), regulatory control deficits (*ρ* = .17, *p* < .001), and internalizing symptoms (*b* = .15, *p* = .004). Internalizing symptoms fully mediated associations with all compulsive behaviors (*b*'s = .05–.16, all *p* ≤ .01). Impaired avoidance learning was modestly associated with greater alcohol use (*b* = −.12, *p* = .03) and gambling (*b* = −.15, *p* = .02) severity. Exploratory analyses showed distinct avoidance patterns mapped onto cognitive (preoccupation, urges) versus behavioral (control, frequency) components of alcohol-related compulsivity.

**Conclusion:**

Habitual avoidance may represent a transdiagnostic behavioral marker of compulsivity. These findings underscore distinct vulnerability pathways across compulsive domains and support the use of remote tasks to phenotype maladaptive avoidance and related emotional dysregulation.

## Introduction

Compulsivity, which involves repetitive patterns of thought and behavior experienced with reduced voluntary control and persisting despite negative consequences (Luigjes et al., 2019), is implicated across psychiatric domains, most notably obsessive-compulsive disorder (OCD), binge-eating disorder (BED), and addiction (Robbins, Banca, & Belin, 2024). Addictions are commonly conceptualized along an impulsivity–compulsivity spectrum, progressing from rash, reward-driven responding to rigid patterns (Everitt & Robbins, 2016). This trajectory has been described across both substance-related (e.g., alcohol use disorder, AUD) and behavioral (e.g., gambling disorder, GD) addictions (Brooks, Lochner, Shoptaw, & Stein, 2017; Demetrovics, van den Brink, Paksi, Horváth, & Maraz, 2022) and may extend to ‘addictive-like’ behaviors not formally classified as disorders, such as binge-watching, where preliminary evidence links loss-of-control and preoccupation to psychosocial impairment (Flayelle et al., 2022; Rubenking & Bracken, 2018; Steins-Loeber, Reiter, Averbeck, Harbarth, & Brand, 2020).

Prevalence estimates underscore the public-health relevance: OCD affects ∼1–3% of the population (Brock, Rizvi, & Hany, 2025), BED ∼1–2% (Keski-Rahkonen, 2021), GD ∼0.1–6% (Tran et al., 2024), and AUD reaching 13% in high-income countries (Glantz et al., 2020). Problematic binge-watching is less well-quantified, though evidence synthesis indicates it is prevalent among young adults (Flayelle et al., 2020). Despite heterogenous symptom profiles, comorbidity is substantial; for example, OCD and substance-related addictions (including AUD) co-occur in 21–36% of cases (Akosile, Tiyatiye, & Akosile, 2025), suggesting shared mechanisms (Burchi, Makris, Lee, Pallanti, & Hollander, 2019; Figee et al., 2016) that may also operate subclinically (Tiego et al., 2019). These observations align with transdiagnostic/dimensional models of compulsivity (Den Ouden et al., 2022), which we adopt here to examine compulsivity across diverse behavioral expressions.

Contemporary accounts describe compulsivity as comprising heterogeneous subconstructs, with at least three partially dissociable yet interacting components: (i) outcome–value insensitivity — failure to adapt choices when outcome values change, expressed in choices based on prior reinforcement; (ii) cognitive-behavioral inflexibility — persistence despite altered contingencies, tasks, or attentional demands (e.g., perseveration or impaired set-shifting); and (iii) urges — intrusive, internally-generated drives to act (Lee, Hoppenbrouwers, & Franken, 2019; Voon & Dalley, 2016). Within instrumental control theory (Dickinson & Balleine, 2002), the former reflects an imbalance between goal-directed control, guided by anticipated outcomes (A–O associations), and habitual control, elicited by antecedent cues via well-rehearsed stimulus–response (S–R) associations, with diminished goal-directed control leaving behavior habitual regardless of adaptive value (Buabang, Donegan, Rafei, & Gillan, 2025). In this framework, maladaptive behaviors such as rituals, binges, or relapse signal lapses in cognitive mechanisms that, under intact functioning, override habitual responding (Gillan, Kosinski, Whelan, Phelps, & Daw, 2016; Robbins & Costa, 2017).

While cognitive deficits have been emphasized, emerging evidence highlights negatively-valenced affective mechanisms in compulsivity (Moreno-Montoya, Olmedo-Córdoba, & Martín-González, 2022; Voon et al., 2020). These mechanisms are central to the allostatic model of addiction, which posits that chronic engagement shifts motivation from positive reinforcement—behavior driven by hedonic reward—to negative reinforcement, where behavior is performed to alleviate escalating distress (Koob & Schulkin, 2019). Similar biases toward distress-avoidance have been reported across compulsive behaviors (Dvorak, Arens, Kuvaas, Williams, & Kilwein, 2013; Flayelle et al., 2022; Litwin, Goldbacher, Cardaciotto, & Gambrel, 2017; Mento et al., 2024; Riley, 2014; Wetterneck, Steinberg, & Hart, 2014), where repeated enactment is thought to disrupt adaptive emotion regulation and contribute to their maintenance (Den Ouden et al., 2020; Kober, 2014).

Despite conceptual overlap, instrumental and affective frameworks are often examined separately, limiting integrative research (Heather, 2017). To address this gap, we propose a transdiagnostic pathway underlying compulsivity involves the amplification of negative affect through habitual avoidance—a process that may sustain compulsive behaviors across clinical and subclinical populations.

### Habitual control in compulsivity

Goal-directed behavior recruits neural systems involved in evaluating motivational salience, whereas habitual control relies on those supporting motor execution (Balleine & O’Doherty, 2010; Watson, van Wingen, & de Wit, 2018). Consistent with dual-systems models, neuroimaging suggests a shift from goal-directed to sensorimotor engagement in compulsivity-related disorders during provocation (Banca et al., 2015; Brevers, Koritzky, Bechara, & Noël, 2015; Sjoerds, Brink, Beekman, Penninx, & Veltman, 2014; Vollstädt-Klein et al., 2010; Wang et al., 2023).

Beyond neurofunctional associations, behavioral assays directly measure which control system predominates in learned behavior. A widely used approach is outcome devaluation, which tests whether behavior adapts to reduced outcome value (Adams & Dickinson, 1981). In rodents, reward devaluation is typically achieved through selective satiety or conditioned aversion, with habits classically induced by overtraining (Robbins & Costa, 2017). Early human translations similarly adopted food-based designs, where responses for snacks subsequently became unrewarding (Valentin, Dickinson, & O’Doherty, 2007). Later adaptations extended this paradigm to assess outcome sensitivity across multiple S-R-O mappings (de Wit, Corlett, Aitken, Dickinson, & Fletcher, 2009) and under conditions requiring rapid choice inhibition (Gillan et al., 2011). Other approaches omit explicit devaluation, instead inferring reliance on goal-directed versus habitual control from sensitivity to probabilistic state transitions (Daw, Gershman, Seymour, Dayan, & Dolan, 2011).

Across paradigms, reduced goal-directed control has been reported in OCD (Gillan et al., 2011; Voon et al., 2015), AUD (Sebold et al., 2014; Sjoerds et al., 2013), BED (Voon et al., 2015), and GD (Wyckmans et al., 2019), often in proportion to disorder severity. Comparable patterns have been observed in non-clinical samples, where reduced goal-directed control correlates with greater severity of alcohol use, binge-eating (Gillan et al., 2016), obsessive-compulsive (OC) symptoms (Gillan et al., 2016; Snorrason & Lee, 2016) and more frequent gambling (Bruder, Wagner, Mathar, & Peters, 2021).

Traits and states may also bias toward habitual behavior. Impulsivity—across multiple subtypes—has been linked to reduced goal-directed control in humans (Dietrich, de Wit, & Horstmann, 2016; Hogarth, Chase, & Baess, 2012) and theorized to accelerate S-R learning by enhancing cue reactivity and weakening A-O encoding (Everitt & Robbins, 2016). Stress similarly promotes habitual responding across species (Schwabe & Wolf, 2009, 2011). Negative emotionality, including low mood and anxiety (Gustavson et al., 2024), has been associated with cognitive inflexibility (Gilbert, Tonge, & Thompson, 2019; Stefanovic, Rosenkranz, Ehring, Watkins, & Takano, 2022) and altered reinforcement learning (Aylward et al., 2019), suggesting another route by which habits may emerge.

### Emotional regulation through avoidance-based mechanisms

In both clinical and community samples, compulsive behavior shows robust associations with negative emotionality, evidenced by frequent co-occurrence of mood and anxiety disorders and elevated internalizing symptom severity (Alimoradi et al., 2022; González-Roz, Castaño, Krotter, Salazar-Cedillo, Gervilla, 2024; Hathaway et al., 2024; Kowalewska, Bzowska, Engel, & Lew-Starowicz, 2024). Neural systems supporting emotion generation and regulation have been implicated (Ochsner, Silvers, & Buhle, 2012), with dysfunction linked to negative attentional bias, rumination, hyperarousal, and altered reward processing (Price & Drevets, 2010; Shin & Liberzon, 2010).

These mechanisms overlap with neurocognitive impairments observed in compulsivity-related disorders: OCD involves threat-related attentional capture, while addictions and binge-eating are characterized by dysregulated reward valuation (from heightened cue reactivity to blunted hedonic response), reduced motivation (apathy), and impaired inhibitory control in affective contexts (Donofry, Roecklein, Wildes, Miller, & Erickson, 2016; Ducharme, Dougherty, & Drevets, 2016; Kober, 2014). In addictions, chronic overstimulation of reward pathways is proposed to induce opponent-process adaptations that intensify negative affect (Koob & Schulkin, 2019), persisting beyond withdrawal and sustaining cravings/urges and preoccupations (Voon et al., 2020). Preoccupation is thought to reflect a cognitive dimension of compulsivity, which—though often co-occurring with behavioral forms—may arise via partly distinct mechanisms (Fineberg et al., 2014; Robbins et al., 2024), and robustly predicts behavioral escalation (Allen & Fillmore, 2023) and relapse (Gilbey & Wilcockson, 2025).

Under this framework, compulsive behaviors represent maladaptive distress-regulation strategies (Den Ouden et al., 2020). Similar to mood and anxiety disorders, compulsivity-related disorders involve greater use of avoidance-based regulation, including suppression and distraction, over adaptive engagement (Aslan, Dorey, Grant, & Chamberlain, 2024). Though these strategies can yield short-term relief, longitudinal studies indicate they seldom resolve emotional conflict and instead exacerbate distress (Spinhoven, Drost, de Rooij, van Hemert, & Penninx, 2014, 2017). Examples include “drinking-to-cope” (Luoma, Pierce, & Levin, 2020) or performing rituals to neutralize intrusive thoughts (Shemrani, Najafi, & Khosravani, 2025), where immediate relief reinforces avoidance despite limited long-term benefit.

### From goal-directed to habitual avoidance

While most instrumental control research emphasizes appetitive learning, a smaller literature examines avoidance—behaviors enacted (active) or withheld (passive) to prevent aversive outcomes. Human paradigms adapted principles from animal models, where Pavlovian threat conditioning (e.g., cue-shock pairing) precedes extended instrumental training (e.g., button-pressing to prevent shock), with avoidance behavior maintained by negative reinforcement. Outcome sensitivity can be tested by reducing or eliminating the aversive consequence (Ball & Gunaydin, 2022). Using shock-based designs, Gillan et al. (2014, 2015) demonstrated that individuals with OCD were more likely to persist in avoidance following overtraining and explicit shock deactivation, consistent with a shift toward habitual control in aversive contexts.

Patterson, Craske, and Knowlton (2019) extended this paradigm by substituting shocks with aversive auditory stimuli (e.g., human screams). Sounds high in acoustic “roughness” reliably elicit strong aversive responses and urgency; empirical work indicates that at moderate intensities they can match or exceed shocks in unpleasantness across self-report and physiological indices (Den, Graham, Newall, & Richardson, 2015; Glenn, Lieberman, & Hajcak, 2012; Neumann et al., 2006, 2008) and accelerate defensive responding (Arnal, Flinker, Kleinschmidt, Giraud, & Poeppel, 2015). This auditory-based approach offers practical advantages, notably compatibility with remote delivery (Seow & Hauser, 2021), thereby broadening accessibility while preserving experimental control.

### The present study

The present study investigated the role of habitual avoidance in compulsivity. Drawing on theories of instrumental control and emotion regulation, we hypothesized that habitual avoidance—indexed by persistence following devaluation—would be associated with compulsive symptom severity across domains, with negative emotionality mediating this relationship. To establish specificity, we also evaluated avoidance learning in relation to compulsivity. Finally, we explored whether associations differed between cognitive (preoccupations) and behavioral (compulsive drinking) symptom dimensions in alcohol use.

## Methods

### Participants

Sample size estimates (G*Power; Faul, Erdfelder, Lang, & Buchner, 2007) assumed 80% power, *α* = .05, and two-tailed tests, yielding a required sample of 110–252 for medium-to-large effects. We recruited 500 UK residents via Prolific (www.prolific.co) for remote participation. Eligibility required English-proficiency, age 18–65, and no psychiatric, neurological, or hearing impairments. To ensure variability in alcohol use, Prolific pre-screening tools stratified recruitment across non-clinical self-reported consumption bands (weekly UK units: ½ pint beer, 1 small wine, or pub measure spirits): 0 = abstinent, 1–4 = low, 5–9 = moderate, 10–13 = high, 14+= hazardous. Demographic data, including gender and education, were also collected.

Of 500 recruited, 443 were retained after quality-control procedures (see Statistical Analysis). The retained sample (M_Age = 43.8, SD = 12.8) was 53.5% male with a mean of 15.3 (SD = 4) years of education.

### Materials and measures

#### Auditory stimuli

Two aversive stimuli were employed: (1) *metal scraping slate* (“scratch”), validated in normative and at-risk cohorts (Neumann et al. (2006, 2008); and (2) *woman's scream* (“scream”), sourced from the International Affective Digitized Sounds Extended (IADS-E; ID = 0276; valence = 2.36 ± 1.18, arousal = 7.05 ± 1.21, dominance = 3.14 ± 1.46) (Yang et al., 2018).

Stimuli were pre-processed in Audacity® 3.0.0, trimmed to 1000 ms, standardized to 44,100 Hz, and converted to mono 32-bit floating point to minimize distortion.

#### Volume calibration and headphone screening

Participants set device volume to 35% of maximum—loud yet comfortable for healthy adults (Seow & Hauser, 2021). Headphones were required to ensure fidelity and reduce external noise, with compliance verified using a validated headphone test (Woods, Siegel, Traer, & McDermott, 2017). The six-item test assessed sensitivity to intensity differences across tones; as phase cancellation impairs speaker accuracy, successful performance (≥5/6 correct) confirmed headphone use. Participants scoring below threshold were excluded. Auditory stimuli were then presented in randomized order.

#### ADT paradigm

**Fig. 1.**
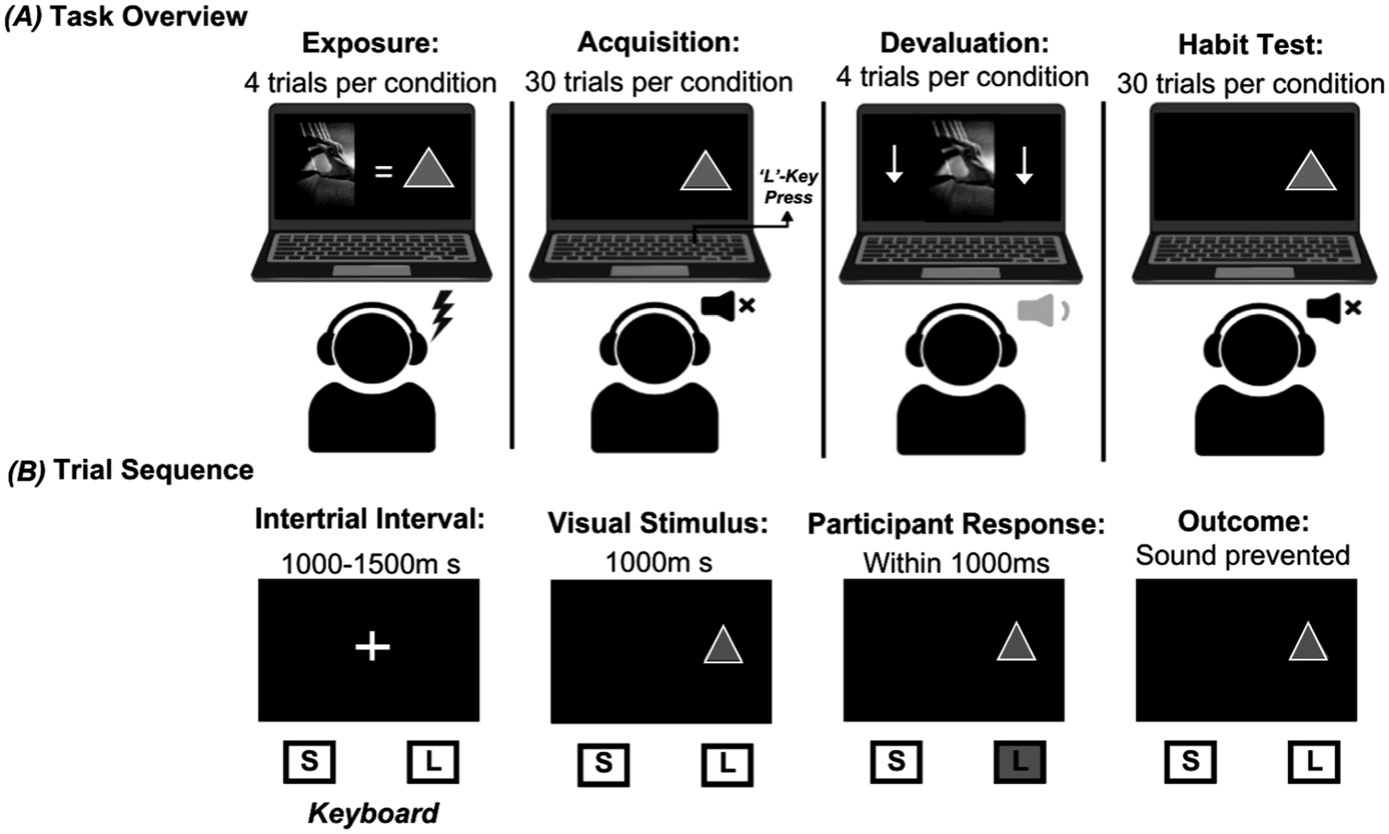
Avoidance Dynamics Task (ADT) paradigm (A) and trial sequence (B). *(A) Task overview*. The ADT included three main phases: acquisition, devaluation, and a habit test. Each auditory outcome (scream, scratch, or silence) was paired with a distinct visual cue. *(B) Trial structure*. Each trial began with an inter-trial interval (1000–1500 ms), followed by cue presentation (1000 ms). Participants had 1000 ms to respond; outcomes were contingent on accuracy

Full task details are provided in Supplementary Materials Section 1 (Fig. 1A). The task was implemented in *PsychoPy2* (Peirce et al., 2019). Before the main task, participants rated two aversive stimuli (scream, scratch) for pleasantness on a 5-point Likert scale (1 = extremely unpleasant, 5 = extremely pleasant), presented once each at full intensity and 15% volume in randomized order.

During *exposure*, each sound and a silent control were paired with a distinct visual cue. During *acquisition*, cues appeared randomly on the left or right side of the screen, and participants pressed the corresponding key (‘S’ or ‘L’) within a 1000 ms window to avoid the associated sound. Correct responses prevented playback; incorrect or missed responses triggered full-intensity sound. Each trial began with an inter-trial interval (1000–1500 ms), followed by cue presentation (1000 ms) (Fig. 1B).

In the *devaluation* phase, the volume of one aversive sound was reduced to 15% while the other remained at full intensity. Finally, the *habit test* was conducted in extinction, with all outcomes withheld; persistent responding to the devalued cue was interpreted as habitual.

*Cue-outcome contingencies* were rated after acquisition and again following the habit test, after which participants also rated their *urge to respond* and *difficulty withholding responses* to devalued cues.

#### Compulsive behavioral measures

- **Alcohol use**: Alcohol Use Disorders Identification Test (AUDIT; Saunders, Aasland, Babor, De La Fuente, & Grant, 1993; 0–40; higher = more hazardous drinking). Obsessive-Compulsive Drinking Scale (OCDS; Anton, 2000; 0–56, comprising obsessional (0–24) and compulsive (0–32) subscales; higher = greater preoccupation with drinking and loss-of-control).
- **Binge eating**: Binge Eating Scale (BES; Gormally, Black, Daston, & Rardin, 1982; 0–46; higher = greater severity).
- **Binge watching**: Binge-Watching Addiction Questionnaire (BWAQ; Forte, Favieri, Tedeschi, & Casagrande, 2021; 0–88; higher = more addictive-like viewing).
- **Gambling**: Gambling Symptom Assessment Scale (G-SAS; Kim, Grant, Potenza, Blanco, & Hollander, 2009; 0–48; higher = greater severity).
- **OC symptoms**: Obsessive-Compulsive Inventory-Revised (OCI-R; Foa, Kozak, Salkovskis, Coles, & Amir, 1998; 0–72; higher = greater severity).

#### Affective and motivational measures

- **Anxiety symptoms:** Generalized Anxiety Disorder Scale (GAD-7; Spitzer, Kroenke, Williams, & Löwe, 2006; 0–21; cut-offs: 5 = mild, 10 = moderate, 15 = severe).
- **Depressive symptoms:** Beck's Depression Inventory Revised (BDI-II; Beck, Ward, Mendelson, Mock, & Erbaugh, 1961; 0–63; higher = greater severity).
- **Anhedonia:** Snaith-Hamilton Pleasure Scale (SHAPS; Snaith et al., 1995; 0–14; higher = lower hedonic tone).
- **Apathy:** Apathy Evaluation Scale Self-Rated (AES-S; Glenn, 2005; 18–72; higher = greater severity).

#### Attentional checks

Each questionnaire included a Likert-scale attention check (e.g., *If you are reading this, please select ‘Strongly Agree’*). Participants scoring <50% were excluded.

### Procedure

**Fig. 2.**
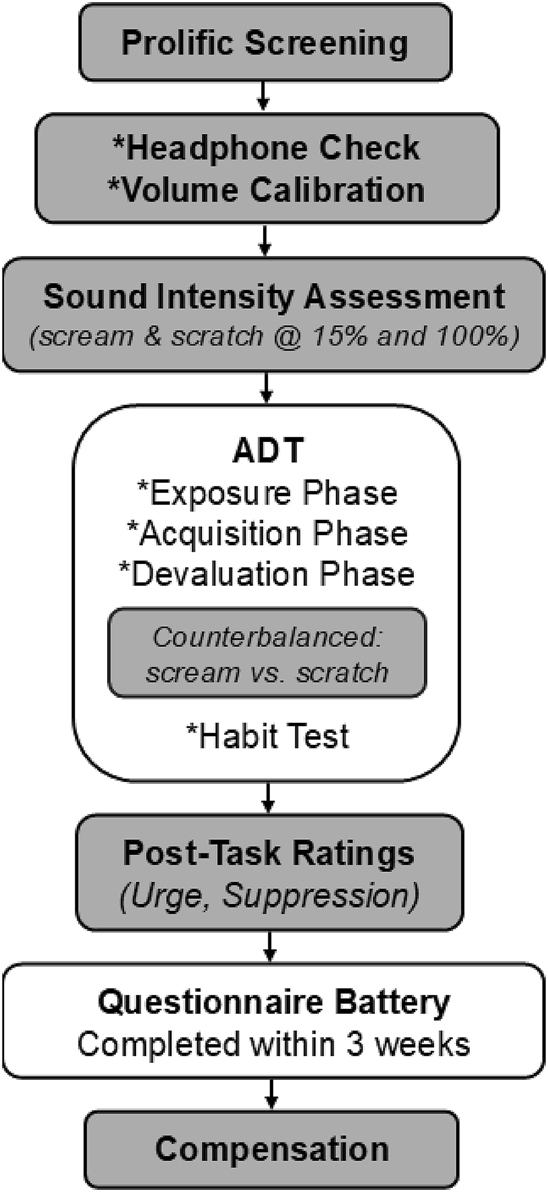
Study procedure timeline. Participants completed sound intensity ratings and the Avoidance Dynamics Task (ADT), followed by post-task ratings. A questionnaire battery was completed within three weeks of task participation

Figure 2 provides a schematic overview of the study timeline. Participants accessed the ADT through unique Prolific links, with devaluation conditions counterbalanced. After volume calibration and headphone screening, they completed the ADT (∼15 min), followed by a randomized questionnaire battery with embedded attentional checks (∼12 min). All procedures were completed within a three-week window. Participants were compensated at £6/h.

### Statistical analysis

Participants were excluded for incomplete tasks (*N* = 24), low headphone scores (*N* = 10), non-responsiveness (*N* = 12), and guideline misunderstandings (*N* = 11), leaving a final analytic sample of *N* = 443. All completed the ADT and questionnaires, except the GSAS and BWAQ (subset *N* = 300). Twenty-two failed questionnaire attention checks, but these overlapped with prior exclusions; no further removals were necessary. Thus, analytic sample sizes varied by model depending on available questionnaire data.

Analyses were conducted in JASP software (Version 0.17.3; 2023). Normality (Shapiro-Wilk) and variance (Levene's) assumptions were assessed, and leverage diagnostics indicated no influential outliers (Belsley, Kuh, & Welsch, 2005). Nonparametric tests validated the ADT with non-normally distributed outcomes (Wilcoxon signed-rank test, Friedman's ANOVA with Conover's post hoc test, chi-square (*X*^2^), and Spearman's rho). The primary task outcome was avoidance habit (responses to devalued cues), with avoidance learning (response accuracy during acquisition) examined in parallel as a secondary outcome.

Two mediation models tested whether negative emotionality mediated associations between (1) avoidance learning and compulsivity and (2) habit and compulsivity (AUDIT, BES, BWAQ, G-SAS, OCI-R). Negative emotionality was operationalized as a linear composite of BDI-II and GAD-7 scores, an approach reflecting their well-documented comorbidity, shared variance (Saha et al., 2020), and treatment in prior work as indicators of a common internalizing factor (Gustavson et al., 2024); this was supported by their strong correlation in our sample (*ρ* = .75, *p* < .001). Covariates included demographics (age, gender, education), motivation (AES; to account for nonspecific motivational deficits [Husain & Roiser, 2018]), and task control (responses to valued stimuli). Significant effects with AUDIT were further examined using OCDS subscales.

Models were estimated using 5000-sample bootstrapping with bias-corrected CIs, and missing data handled via FIML (Biesanz, Falk, & Savalei, 2010). Robustness checks included alternative mediators (SHAPS, to test specificity against reward-processing impairments; BDI-II and GAD-7 separately), reversed predictor–mediator models, split-half validation, and specificity tests with valued-stimulus responses. Significance was set at FDR-corrected *p* < .05 (Benjamini & Hochberg, 1995), with 95% CIs reported.

### Ethics

Procedures were approved by the Cambridge Psychology Research Ethics Committee and conducted in accordance with the Declaration of Helsinki (2008) and relevant institutional and national guidelines. All participants provided informed consent.

## Results

### Demographic information

Table 1 presents participant demographics and internal consistency values (Cronbach's *α*) for all questionnaire measures.

**Table 1.** Internal consistency measures and sample characteristics

Factor		*α*	Mean/*N*	SD/*%*	Min	Max
*Covariates*						
Demographic *N = 443*	Age		43.8	12.8	18	77
	Gender (*M*)		*237*	*53.5%*		
	Years of education		15.3	4	5	26
Mood *N = 443*	Depression (*BDI-II*)	.94	12	10.9	0	54
	Anxiety *(GAD-7)*	.94	4.4	4.9	0	21
Motivation *N = 443*	Anhedonia (*SHAPS*)	.9	22	6.9	5	42
	Apathy (*AES-S*)	.87	48.2	7.9	32	72
*Outcomes*						
Alcohol Use *N = 443*	General *(AUDIT);*	.85	7	5.7	0	35
	Preoccupations (*OCDS-O*)	.78	1	2	0	11
	Compulsions (*OCDS-C*)	.79	3.2	3.2	0	16
Binge-Eating (*BES*); *N = 443*		.84	9.6	8.7	0	43
Binge-Watching (*BWAQ*); *N = 300*		.95	24.5	11.8	0	80
Gambling *(G-SAS); N = 300*		.89	6.8	7.6	0	39
OC Symptoms (*OCI-R*); *N = 443*		.88	11.5	7.7	0	45
OC Symptom Subtypes	Checking	.79	2.3	6.6	0	12
	Hoarding	.71	2.4	2.3	0	12
	Neutralizing	.76	1.3	2.1	0	12
	Obsessing	.88	2	2.7	0	12
	Ordering	.89	2.5	2.6	0	12
	Washing	.81	1.1	1.8	0	11

*Note:* Internal consistency was assessed using Cronbach's alpha (*α*), indicating that questionnaires exhibited internal consistency ranging from acceptable to excellent. For quantitative variables, including demographic (age, years of education), mood (depression [BDI-II], anxiety [GAD-7]), and motivational (anhedonia [SHAPS], apathy [AES]) variables, as well as outcomes (general alcohol use [AUDIT], drinking obsessions [OCDS-O], drinking compulsions [OCDS-C], binge eating [BES], binge watching [BWAQ], gambling [G-SAS], and OC symptoms [OCI-R] with subscales for checking, hoarding, neutralizing, obsessing, ordering, and washing), the mean, standard deviation (SD), and minimum (Min) and maximum (Max) values are reported. For gender, frequencies are reported.

### ADT validation

**Fig. 3.**
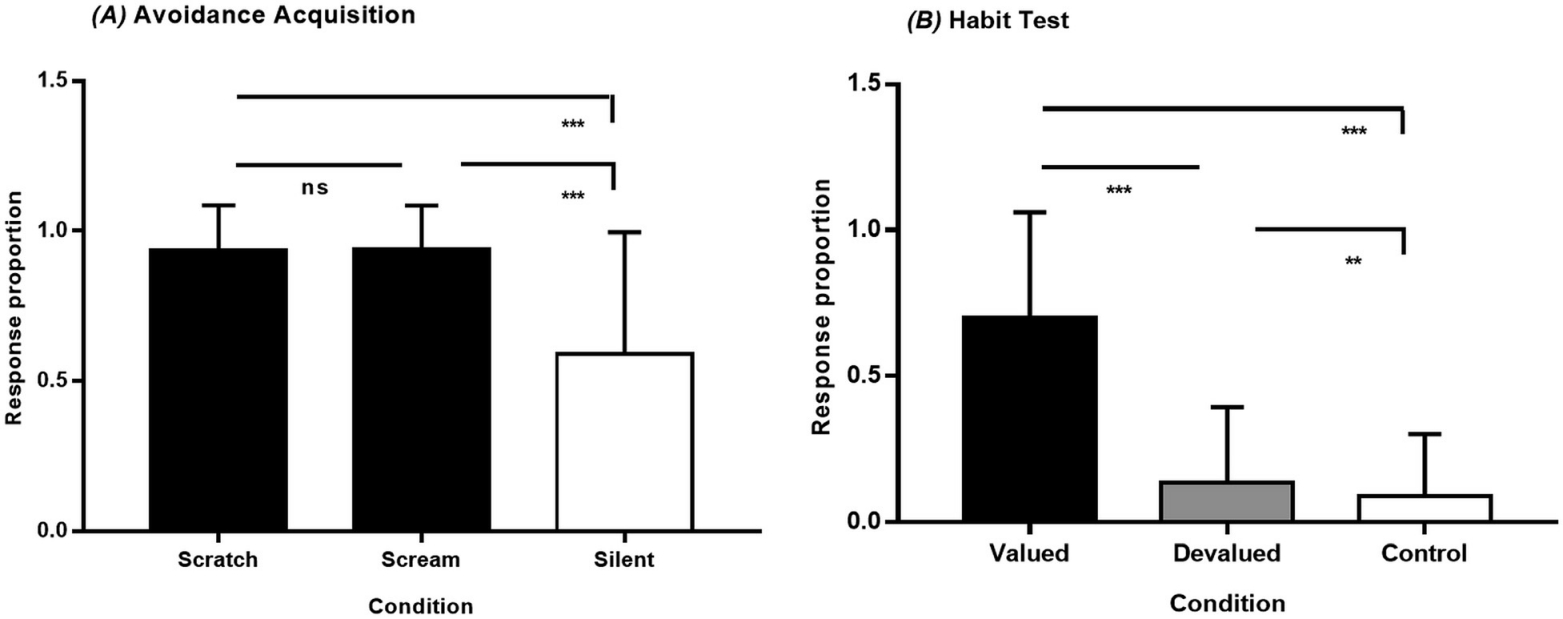
Mean response rates to each visual cue during (A) the acquisition phase and (B) the habit test of the Avoidance Dynamics Task (ADT). Error bars represent standard deviations. FDR-corrected *p*-value = *< .05, **< .01, ***< .001

Participants rated both sounds as less pleasant at maximum versus low volume (scream: *W* = 1,355, *p* < .001, CIs: −.84 to −.67; scratch: *W* = 1119.5, *p* < .001, CIs: −.83 to −.7), confirming affective modulation. During acquisition, response rates to both active stimuli were comparable (*p* > .05) and significantly higher than to the silent control (*X*^2^ = 223.8, *p* < .001; Fig. 3A), indicating reliable cue-outcome learning. Response rates were correlated negatively with pleasantness (*ρ* = −.21, *p* = .009) and positively—though weakly—with arousal (*ρ* = .11, *p* = .01). RTs did not differ by condition (all *p* > .05).

In the habit test, 237 of the 443 participants showed completed devaluation (score = 0). Across the full sample, responses to devalued stimuli were lower than to valued stimuli (*t* = 15.5, *p* < .001) and higher than to control stimuli (*t* = 3.5, *p* = .002; Fig. 3B). This gradient (valued > devalued > control) suggests outcome sensitivity alongside residual outcome-insensitive responding, consistent with partial engagement of S–R mechanisms and supporting ADT sensitivity to both goal-directed and habitual components of avoidance behavior (Patterson et al., 2019).

Devalued responses were unrelated to contingency knowledge or valence/arousal ratings (all *p* > .05) but positively correlated with urge to respond (*ρ* = .28, *p* < .001) and difficulty suppressing responses (*ρ* = .17, *p* < .001), supporting convergent validity with self-reported motivational conflict.

Further validation analyses are in Supplemental Materials, Section 2.

### Model 1: avoidance learning

**Fig. 4.**
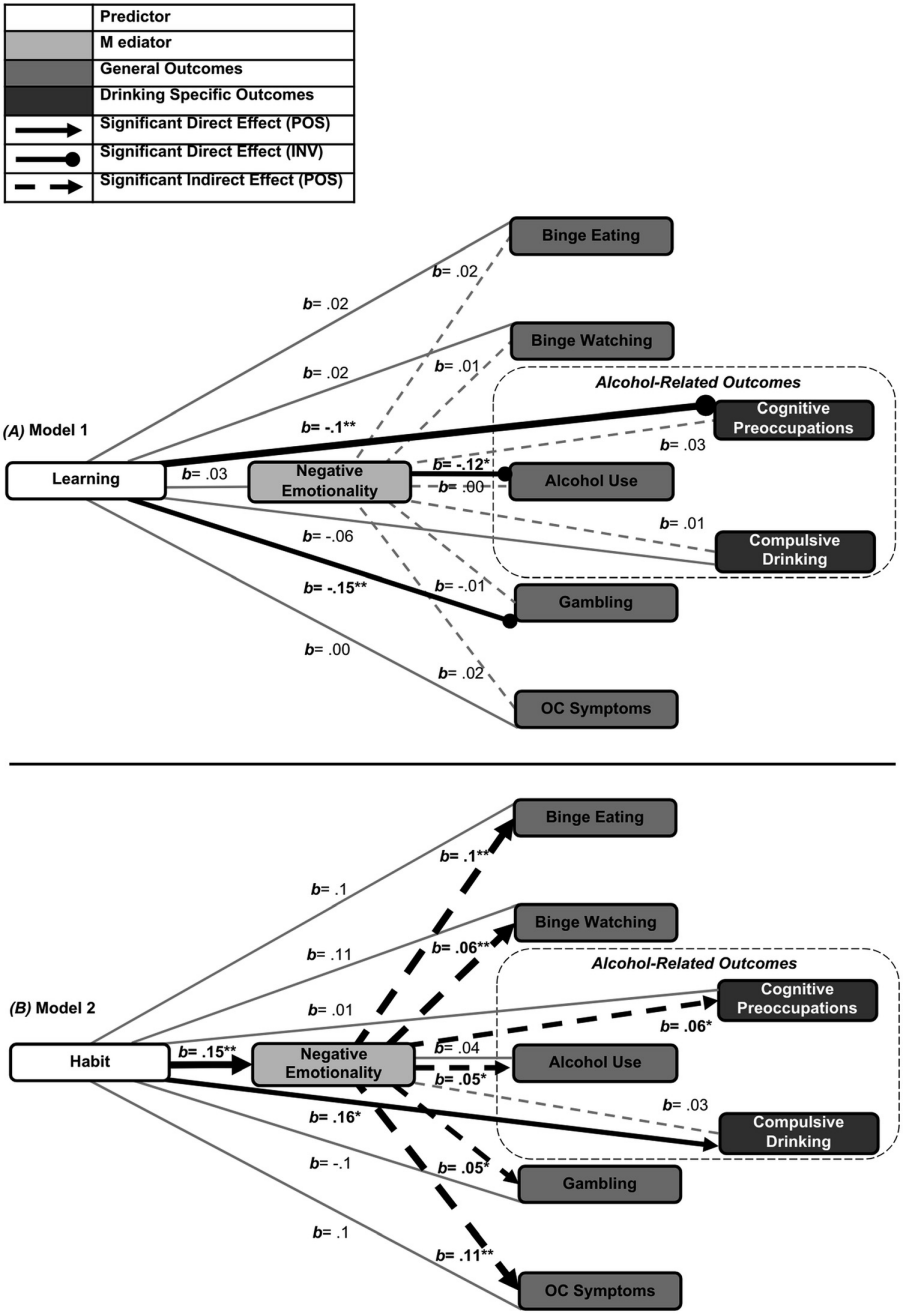
Mediation model results for (A) avoidance learning and (B) habitual avoidance. Each panel includes a primary model (targeting general compulsive behaviors) and an exploratory model (targeting alcohol-specific outcomes: cognitive preoccupations and compulsive drinking). Solid black lines indicate significant direct effects; dashed lines indicate significant indirect (mediated) paths via negative emotionality. Line thickness corresponds to standardized beta weights (relationship magnitude). All models control for age, gender, years of education, and apathy (AES-S scores); Panel B models additionally control for response rates to valued cues post-devaluation. FDR-corrected *p*-value: *p < .05, **p < .01, ***p < .001

Avoidance learning responses were inversely associated with alcohol use severity (*b* = −0.12, *p* = .03; CIs: −1.03 to −0.01; *R*^2^ = .10) and gambling severity (*b* = −0.15, *p* = .02; CIs: −1.37 to −0.11; *R*^2^ = .10; Fig. 4A), accounting for modest variance. No indirect effects via negative emotionality were observed (all *p* > .05).

#### Avoidance learning and OCDS subscales

Avoidance learning responses were inversely associated with preoccupation-related drinking symptom severity (*b* = −0.2, *p* = .002; CIs: −1.68 to −0.37; *R*^2^ = .13, Fig. 4A), but not compulsive drinking symptom severity (*p* > .05).

### Model 2: avoidance habit

Avoidance habit strength showed no direct effects on compulsive behavior severity, but was positively associated with negative emotionality (*b* = .15, *p* = .004; CIs: 1.02 to 6.27, *R*^2^ = .4), which significantly mediated all modeled indirect pathways (Table 2, Fig. 4B).

**Table 2.** Indirect effects and *R*-squared values of Model 2: Avoidance habit

Compulsivity domain	Indirect effect (b)	*p* value	Confidence intervals (CIs)	Model *R*^2^
Alcohol Use *(AUDIT)*	0.05	0.01*	0.22 to 1.55	0.11
Binge Eating *(BES)*	0.10	0.005**	0.6 to 4.09	0.42
Binge Watching *(BWAQ)*	0.06	0.008**	0.61 to 3.58	0.23
Gambling *(G-SAS)*	0.05	0.01*	0.38 to 2.39	0.16
Obsessive-Compulsive *(OCI-R)*	0.11	0.004**	0.92 to 5.5	0.41

*Note:* This table presents the indirect effects of avoidance habits on symptom severity across compulsive domains, mediated by negative emotionality, along with their respective standardized estimates (b), *p*-values, confidence intervals (CIs), and *R*-squared (*R*^2^) values. *R*^2^ values indicate the proportion of variance explained by the full mediation model for each outcome variable. FDR-corrected *p*-value = *< .05, ** < .01, *** < .001.

Model *R*^2^ values were highest for binge eating symptom severity (*R*^2^ = .42) and OC symptom severity (*R*^2^ = .41), followed by binge watching severity (*R*^2^ = .23), gambling severity (*R*^2^ = .16), and alcohol use severity (*R*^2^ = .11).

Total effects were significant for binge eating (*b* = .2, *p* = .001, CIs: 1.5 to 8) and binge watching (*b* = .16, *p* = .01, CIs: 1.45 to 9.44) symptom severities.

Findings held when including anhedonic symptom severity as an additional mediator (Supplementary 3.1), in split-halved samples (Supplementary 3.2), and when depressive and anxiety symptom severities were entered as separate mediators (Supplementary 3.3), but not under reversed model direction (Supplementary 3.4) or with valued cue responses as predictors (Supplementary 3.5).

#### Avoidance habits and OCDS subscales

Avoidance habit was directly associated with compulsive drinking symptom severity (*b* = .16, *p* = .03; CI: .01 to .31; *R*^2^ = .05; Fig. 4B), though the effect was small. No indirect effect via negative emotionality emerged (*p* > .05). For drinking preoccupation symptom severity, negative emotionality weakly mediated the relationship (*b* = .06, *p* = .01; CI: .01 to .11; *R*^2^ = .12; Fig. 4B); no direct or total effects were significant (both *p* > .05).

## Discussion

Using an aversive devaluation task in a non-clinical sample, we examined dimensional links between avoidance habits and compulsive behaviors across alcohol use, binge-eating, binge-watching, gambling, and OC symptoms. We expected stronger avoidance habits to relate to compulsive symptom severity via negative emotionality and ran parallel models with avoidance learning to contextualize these effects. Finally, we explored distinct associations for cognitive and behavioral components of alcohol use.

### Avoidance Dynamics Task (ADT) as an online measure of avoidance habits

To our knowledge, this study is the first to assess avoidance instrumental control in an online setting. The Avoidance Dynamics Task (ADT) incorporated established overtraining and devaluation procedures and showed robust devaluation effects, consistent with prior work (Dietrich et al., 2016; Gillan et al., 2014, 2015; Snorrason & Lee, 2016). Nevertheless, nearly half of participants persisted in responding to devalued cues following contingency overtraining—independent of learning, explicit awareness, or valuation sensitivity. Instead, habitual response patterns correlated with urges to respond to devalued cues and difficulty suppressing them, consistent with cue-elicited S-R behavior (Robbins & Costa, 2017) linked to compulsive urges (Everitt & Robbins, 2016); see also Millner, Gershman, Nock, and den Ouden (2018) for Pavlovian contributions).

Our approach diverges from prior threat acquisition and habit studies in rodents and humans which used shock-based reinforcers (Ball & Gunaydin, 2022; Gillan et al., 2014, 2015) by employing affectively salient auditory stimuli (see also Patterson et al., 2019). Ratings of low pleasantness and high arousal were associated with near-maximal avoidance during acquisition, indicating these stimuli effectively motivated behavior and may promote conditions for habit formation, as rapid, repetitive responding is thought to foster reliance on habits. This extends classic habit procedures (Adams & Dickinson, 1981) to an online format, enabling scalable human testing while retaining continuity with animal models.

While the ADT isolates habitual responding through devaluation, it does not capture arbitration during ongoing choice—processes implicated in compulsive phenomena (Gillan et al., 2016; Voon et al., 2015). Aversive two-step tasks could complement the ADT by quantifying flexibility, exploration, and arbitration under threat, thereby delineating distinct facets of avoidance control (Geramita, Yttri, & Ahmari, 2020).

### Impaired avoidance learning in alcohol use and gambling: roles for reward-loss sensitivity and cognitive control

Both excessive and insufficient avoidance may undermine adaptive functioning (Dymond, 2019; Verdejo-Garcia & Albein-Urios, 2021). Here, reduced responding to threat cues during acquisition—reflecting insufficient avoidance—was modestly associated with greater alcohol use severity, particularly cognitive preoccupations with drinking, and gambling severity. These findings point to altered reward-loss sensitivity, alongside deficits in cognitive control—mechanisms implicated in addictive behaviors and their progression toward compulsivity (Leeman & Potenza, 2012).

In alcohol use and gambling, heightened salience attribution in cue-reactivity paradigms and reduced loss aversion in risk–loss decision tasks have been reported (Genauck et al., 2017; Linnet, Møller, Peterson, Gjedde, & Doudet, 2011; Thrailkill, DeSarno, & Higgins, 2022; Vollstädt-Klein et al., 2010). Altered valuation processes may blunt the deterrent value of aversive outcomes and increase demands on cognitive control (Goldstein & Volkow, 2011). In alcohol use, this may hinder suppression of alcohol-related thoughts, which often precede drinking episodes (Allen & Fillmore, 2023; Gilbey & Wilcockson, 2025). Parallel dynamics have been observed in gambling, where regulatory failures are thought to promote loss-chasing (Zhang & Clark, 2020).

These processes converge on impulsivity, a well-established risk factor for compulsive engagement (Verdejo-Garcia & Albein-Urios, 2021; Wypych & Potenza, 2021). In addictive behaviors, impulsive traits such as novelty-seeking despite risk and acting without forethought have been reported across both clinical and community samples (Chowdhury, Livesey, Blaszczynski, & Harris, 2017; Miller & Gizer, 2024; Sliedrecht, Roozen, Witkiewitz, de Waart, & Dom, 2021), and are linked to both blunted punishment sensitivity and habit biases in appetitive contexts (Dietrich et al., 2016; Hirmas & Engelmann, 2023; Hogarth et al., 2012; Xu et al., 2019; Zheng, Tian, Li, & Liu, 2019), consistent with etiological models of addiction in which impulsivity progresses to compulsivity via habit learning (Everitt & Robbins, 2016). By contrast, binge behaviors have been associated with both elevated impulsivity and harm avoidance (Flayelle et al., 2022; Forte, Favieri, Casagrande, & Tambelli, 2023; Gat-Lazer, Geva, Gur, & Stein, 2017), which may preserve avoidance learning. Future work should clarify how divergent impulsivity profiles influence the transition from goal-directed to habitual control under aversive motivation.

### Negative emotionality: a bridge from habit to compulsion

As anticipated, habitual avoidance—defined as persistent responding to devalued threat cues— showed small but statistically significant associations with all measured compulsive behaviors, with negative emotionality accounting for these associations. This supports a possible transdiagnostic role of avoidance habits in compulsivity, with negative emotionality providing a reinforcing context that sustains them.

Habitual, rather than general, avoidance was uniquely associated with internalizing symptoms. This accords with evidence that negative affect impairs cognitive control (Gilbert et al., 2019; Stefanovic et al., 2022) and promotes cognitive and behavioral compulsivity (Fineberg et al., 2014). Convergent experimental and observational studies report heightened attentional bias and perseveration under negative mood induction and in subclinical depression (Bellaera & von Mühlenen, 2017), impaired response inhibition in individuals with low mood and emotional regulation difficulties (King, 2020), and stronger habit learning following early adversity (Patterson et al., 2019) and stress induction (Schwabe & Wolf, 2009, 2011).

Negative affect may therefore erode regulation of previously adaptive avoidance, promoting compulsive engagement across domains. Ecological momentary assessment (EMA) studies support this view: negative affect often precedes binge-eating episodes (Haedt-Matt & Keel, 2011), OC symptom escalation (Goldberg et al., 2016), relapse (Dvorak, Pearson, & Day, 2014), and loss-chasing (Gee, Coventry, & Birkenhead, 2005). Similar dynamics appear in binge-watching, where post-episode anxiety precedes continued viewing (Panda & Pandey, 2017).

Mediation models explained the most variance in binge-eating and OC symptoms, consistent with frameworks implicating executive dysfunction, inhibitory control deficits, and cognitive-behavioral inflexibility, alongside heightened emotional reactivity, in these conditions (Hezel & McNally, 2016; Kober & Boswell, 2018; Thorsen et al., 2018; Voon, 2015). Evidence for binge-watching is more limited, though recent studies suggest two pathways to functional impairment (Steins-Loeber et al., 2020): one impulsivity-driven and one depressive, characterized by coping-based motivations and post-episode distress (Castro, Rigby, Cabral, & Nisi, 2021; Flayelle et al., 2020; Panda & Pandey, 2017). Reports of blunted gain-loss sensitivity and motor disinhibition in high loss-of-control binge-watching (Dieterich, Wüllhorst, Berghäuser, Overmeyer, & Endrass, 2021) further point to a possible role for habitual processes. Our findings may help bridge these accounts: habitual avoidance may constrain emotional processing, thereby prolonging distress and sustaining engagement.

By contrast, poorer model fit for alcohol use and gambling in this nonclinical sample aligns with accounts positioning compulsivity and heightened negative affect as later-stage developments in addictions (Everitt & Robbins, 2016; Koob & Schulkin, 2019). In AUD, goal-directed control appears to normalize with abstinence (Voon et al., 2015), suggesting habit bias emerges through escalation and chronicity rather than contributing to acquisition. Our findings further indicate differentiated pathways to compulsivity in alcohol use: habitual avoidance was modestly—though directly—associated with compulsive drinking (control, frequency) and indirectly to cognitive preoccupations via negative emotionality. While the former aligns with traditional accounts of habit as sensorimotor routines (Buabang et al., 2025; Gillan et al., 2016), the latter suggests habitual avoidance may also translate unresolved emotional conflict into compulsive thought and behavior—extending dual-systems models to account for this affective function.

Clinically, these findings highlight the need to target both cognitive and affective mechanisms in compulsivity. Behavioral Activation, which promotes goal-directed action through structured engagement in valued activities (Martell, Dimidjian, & Herman-Dunn, 2010), may help counter habitual avoidance. Neuromodulation approaches, such as repetitive transcranial magnetic stimulation (rTMS), show promise for enhancing cognitive control and reducing compulsive symptoms across OCD, substance use, and gambling (Del Mauro et al., 2023; Kar, Agrawal, Silva-Dos-Santos, Gupta, & Deng, 2024; Mehta et al., 2024). Future research should prioritize mechanism-informed interventions to address avoidance-driven compulsivity.

### Limitations

Several limitations warrant consideration. Online administration restricted control over compliance variables such as headphone use and volume. While we employed a validated headphone check (Woods et al., 2017) and initial calibration (Seow & Hauser, 2021), within-task verifications could further improve adherence. Our overtraining approach, though effective in rodents (Adams & Dickinson, 1981), may be less robust in humans (de Wit et al., 2018), and absence of early learning assessments limits conclusions about whether observed behaviors reflected goal-directed impairment or habit acquisition. The ADT models avoidance under controlled conditions but may not capture features of real-world threats, such as emotional salience (Phelps & LeDoux, 2005), unpredictability (Grupe & Nitschke, 2013), or cognitive load (Kool & Botvinick, 2018). Ceiling effects during the habit test suggest successful devaluation but may have constrained response variability, potentially contributing to modest effect sizes and small-magnitude associations in regression and mediation models (Uttl, 2005). These associations may therefore hold limited practical significance and should be interpreted cautiously. Longitudinal designs and methods promoting greater behavioral variability will be important for evaluating robustness and generalizability. Finally, although habit bias in aversive contexts has been linked to OCD (Gillan et al., 2014, 2015), the ADT has yet to be applied in AUD, GD, or binge-type disorders. Broader clinical sampling is needed to establish translational relevance.

## Conclusions

This study advances understanding of how avoidance dynamics contribute to compulsivity across transdiagnostic symptom domains. Findings highlight the heterogeneity of compulsive behaviors and the importance of interventions targeting both cognitive and affective dysregulation. By identifying potential early neurocognitive risk markers, this work provides a foundation for translational efforts toward precision therapies targeting avoidance-driven compulsivity.

## Supplementary material

## Data Availability

Deidentified participant data are available from the corresponding author on reasonable request.
