# Supplementary material for: Mapping affective pathways to compulsion: Insights from an aversive devaluation approach
Source: J Behav Addict. 2025 Nov 19;14(4):1517–32. doi: 10.1556/2006.2025.00089 (PMC12767598; doi:10.1556/2006.2025.00089)
Supplement: Supplementary file 1 [file jba-14-1517-s001.pdf]

**Sallie, S.N. et al.: Mapping affective pathways to compulsion: Insights from an aversive devaluation approach.**

https://doi.org/10.1556/2006.2025.00089

# SUPPLEMENTARY MATERIALS

## Section 1: Avoidance Dynamics Task (ADT) Structure

The ADT was developed using PsychoPy3 (Pierce et al., 2007). Generic, colored shapes (10x10 cm) were displayed against a black background for 1000ms. The set included three stimuli: two paired with auditory stimuli (active conditions) and one without sound (control condition), all with deterministic contingencies. The presentation sequence was randomized.

To avoid sound exposure, participants quickly pressed the 'S' key for stimuli on the left and the 'L' key for stimuli on the right using their pinkie fingers. Visual stimuli, paired with auditory cues, were randomly presented on the left or right side of the screen (Figure 1). A correct response, pressing the corresponding key within 1000ms, prevented the auditory stimulus but did not terminate the visual stimulus, which remained for an additional 1000ms. Incorrect, late, or missed responses resulted in sound exposure, with the sound type depending on the visual stimulus. Intertrial intervals varied between 1000-1500ms.

The task comprised the following phases. In the first phase, participants learned stimulus–outcome (S–O) associations, with each visual stimulus explicitly paired with either a sound or silence. They were then instructed that pressing the correct key ('S' for left, 'L' for right) within 1000ms of a visual cue could prevent the auditory stimulus. Participants practiced these responses in four trials per condition before the main experiment.

The experimental protocol comprised three phases: acquisition (30 trials per condition), devaluation (4 trials per condition), and habit test in extinction (30 trials per condition). During devaluation, participants were re-exposed to the auditory stimuli, with one sound's intensity reduced to 15% of its original level, making it nearly inaudible. Participants were informed that responses to avoid these devalued sounds were unnecessary. Devaluation was balanced across participants, with approximately half (N=274) experiencing the scrape sound devalued and the other half (N=226) the scream sound. The intensity of the still-valued sound remained unchanged.

In the habit test, participants responded to visual cues in extinction, where no auditory stimuli were delivered. Unaware that the cues no longer predicted auditory outcomes, participants'

continued responses to cues associated with devalued sounds were taken as evidence of habitual responding.

After the explicit association phase, participants evaluated the pleasantness of both auditory stimuli on a scale from 1 (Extremely unpleasant) to 5 (Extremely pleasant) and assessed their arousal post-exposure on a scale from 1 (Not arousing at all) to 5 (Extremely arousing). Following both the acquisition and habit test phases, contingency knowledge of shape-sound pairings was rated with options 1 (Definitely no), 2 (Unsure), and 3 (Definitely yes). Post-task, participants retrospectively reported their urge to respond to the devalued visual stimulus during the habit test on a 5-point Likert scale (1=No urge to 5=Extreme urge) and their effort to suppress this urge (1=No effort to 5=Extreme effort).

## Section 2: Analysis Strategy and Validation of the Avoidance Dynamics Task (ADT)

*Analysis strategy*

Our first objective was to validate the efficacy of the auditory stimuli in evoking threat responses based on their affective properties. To achieve this, we (1) compared self-reported valence and arousal across different sound intensities, (2) examined response rates to sound-associated visual stimuli relative to silent stimuli during acquisition, and (3) assessed the correlation between response rates and subjective valence and arousal ratings. Task proficiency was assessed via response rates to sound-associated stimuli, supported by response time analysis. Efficacy of the outcome devaluation procedure was examined by contrasting responses toward devalued versus valued cues during the habit test. Furthermore, to affirm S-R habit learning, responses to devalued cues were compared with (1) silent control responses, (2) contingency knowledge, and (3) urge intensity and suppression difficulty. The analysis accounted for the counterbalancing of devalued stimuli, wherein approximately half of the participants completed the task with the scream sound devalued and the other half with the scratch sound devalued, mitigating potential stimulus-specific biases and ensuring that effects were not driven by idiosyncratic aversiveness of a particular auditory cue.

*Sound intensity, pleasantness, and arousal ratings*

No significant differences were observed between the auditory stimuli when presented at full volume or when modulated to 15% of maximum ($p > .05$). However, intra-sound analyses demonstrated significant differences for the scratch sound between the maximum (1.3±.47) and low (2±.7) volumes ($W=1355$, $p < .001$, CI: -.84 - -.67) and for the scream sound between maximum (1.2±.67) and low (2±.44) volumes ($W=1119.5$, $p < .001$, CI: -.83 - -.7).

Following the explicit association phase, participants indicated generally low pleasantness (2.1±.85) and high arousal (4±.7) toward both auditory stimuli.

*Acquisition and habit test response comparison*

During acquisition, response amounts toward visual stimuli associated with either auditory stimulus (active 1: .94±.15 active 2: .94±.16) were not significantly different ($p > .05$), although response amounts in these active conditions were significantly greater than the silent control condition (.59±.41; $X^2$=223.8, $p < .001$). Response rates were inversely associated with pleasantness ratings (ρ=-.21, p=.009) and positively associated with arousal ratings (ρ=.11, p=.01) following explicit association. Average RTs toward all visual stimuli were not significantly different ($p > .05$), suggesting uniform learning rates across conditions.

During the habit test, responses toward the devalued stimulus (.22±.36) were significantly lower compared to responses to the still-valued stimulus (.62±.4, t=15.5, $p < .001$), but exceeded those directed towards the control stimulus (.12±.26, t=3.5, p= .002). Upon assessing condition*devaluation group (0=scream, 1=scratch), we found no significant difference in rate of habit responding based on sound type ($p > .05$). Responses to devalued stimuli were unrelated to subjective pleasantness or arousal ratings (all $p > .05$).

*Explicit contingency ratings*

Following acquisition, explicit contingency ratings did not significantly differ across active conditions ($p > .05$) but exceeded ratings for the silent condition ($X^2$=784.84, $p < .001$). Conversely, post-habit test, ratings for the devalued stimulus were significantly lower than for the valued stimulus (t=11.5, $p < .001$, CI: -.9 - -.62), with no significant difference from the control stimulus rating ($p > .05$). There was no significant correlation between habitual responding and explicit knowledge in any condition, indicating intact contingency knowledge ($p > .05$).

*Response urge and suppression difficulty ratings*

During the habit test, moderate urges to engage with the devalued stimulus were reported (2.05±1.1) alongside suppression difficulties (avoidance: 2.14±1.2). Habitual responding was positively correlated with both urge intensity (ρ=.28, $p < .001$) and suppression difficulty (avoidance: ρ=.17, $p < .001$).

**Section 3: Validation of Indirect Effects in Model 2: Avoidance Habit Mediation Analysis**

*3.1: Inclusion of Anhedonia as an Additional Mediator to Establish the Specificity of Negative Emotionality Effects*

To test whether the association between avoidance habit and compulsivity is specifically driven by elevated negative affect, we conducted a supplementary mediation analysis including both negative emotionality (indexed by linearly combined depression [BDI-II] and anxiety [GAD-7] scores) and anhedonia (SHAPS scores) as parallel mediators. Avoidance habit served as the predictor, with severity of compulsive behaviors across domains as outcome variables. Demographic variables (age, gender, years of education), apathy (AES scores), and responses to still-valued cues post-devaluation were included as covariates (Table 2.1).

Results indicated that negative emotionality significantly mediated the relationship between avoidance habit and severity of compulsive behaviors, while anhedonia did not. The lack of a significant indirect effect via anhedonia suggests that this relationship is not attributable to a general blunting of reward sensitivity but rather reflects a more specific role of elevated negative affect in avoidance-driven compulsivity.

| **Compulsivity Domain** | **Mediator** | **(b)** | **p-value** | **CIs** |
|---|---|---|---|---|
| Alcohol Use *(AUDIT)* | Negative Emotionality | 0.05 | 0.01* | 0.22 to 1.55 |
| | Anhedonia *(SHAPS)* | .001 | 0.77 | -0.1 to 0.25 |
| Binge Eating *(BES)* | Negative Emotionality | 0.10 | 0.005** | 0.6 to 4.09 |
| | Anhedonia *(SHAPS)* | .0001 | 0.77 | -0.11 to 0.36 |
| Binge Watching *(BWAQ)* | Negative Emotionality | 0.06 | 0.008** | 0.61 to 3.58 |
| | Anhedonia *(SHAPS)* | .001 | 0.78 | -0.19 to 0.52 |
| Gambling *(G-SAS)* | Negative Emotionality | 0.05 | 0.01* | 0.38 to 2.39 |
| | Anhedonia *(SHAPS)* | .0001 | 0.83 | -0.29 to 0.12 |
| Obsessive-Compulsive *(OCI-R)* | Negative Emotionality | 0.11 | 0.004** | 0.92 to 5.5 |
| | Anhedonia *(SHAPS)* | -.001 | 0.77 | -0.52 to 0.18 |

**Table 3.1**. Inclusion of Anhedonia as an Additional Mediator to Establish the Specificity of Negative Emotionality Effects

*3.2: Robustness Check of Mediation Analysis Using Split-Half Sampling*

To assess the robustness of the mediation model, we conducted a split-half validation using the odd/even method, assigning participants to one of two subgroups based on whether their participant ID was odd or even (Odd: Table 2.2.a; Even: Table 2.2.b).

Mediation analyses were then repeated separately within each subgroup. The pattern of effects observed in the full sample was replicated across both subgroups, supporting the stability and generalizability of the mediation findings. This consistency suggests that the results are not driven by sample-specific variability.

| **Compulsivity Domain** | **Indirect Effect (b)** | **p-value** | **Confidence Intervals (CIs)** | **Model $R^2$** |
|---|---|---|---|---|
| Alcohol Use *(AUDIT)* | 0.05 | 0.02* | 0.02 to 0.12 | 0.1 |
| Binge Eating *(BES)* | 0.10 | 0.005** | 0.03 to 0.19 | 0.41 |
| Binge Watching *(BWAQ)* | 0.07 | 0.01** | 0.03 to 0.14 | 0.25 |
| Gambling *(G-SAS)* | 0.06 | 0.02* | 0.02 to 0.14 | 0.11 |
| Obsessive-Compulsive *(OCI-R)* | 0.13 | 0.004** | 0.04 to 0.23 | 0.46 |

**Table 3.2.a**: Even-ID Group

| **Compulsivity Domain** | **Indirect Effect (b)** | **p-value** | **Confidence Intervals (CIs)** | **Model $R^2$** |
|---|---|---|---|---|
| Alcohol Use *(AUDIT)* | 0.04 | 0.04* | 0.01 to 0.08 | 0.11 |
| Binge Eating *(BES)* | 0.08 | 0.02* | 0.01 to 0.15 | 0.41 |
| Binge Watching *(BWAQ)* | 0.05 | 0.03* | 0.003 to 0.1 | 0.23 |
| Gambling *(G-SAS)* | 0.06 | 0.04* | 0.002 to 0.12 | 0.21 |
| Obsessive-Compulsive *(OCI-R)* | 0.1 | 0.03* | 0.01 to 0.17 | 0.4 |

**Table 3.2.b**: Odd-ID Group

### *3.3: Disaggregated Mediation Models for Depressive (BDI-II) and Anxiety (GAD-7) Symptoms*

To further assess the robustness and specificity of the composite negative emotionality construct, we conducted additional mediation analyses for Model 2 using depression (BDI-II) and anxiety (GAD-7) scores as separate mediators. These models were structured identically to the main analysis, examining indirect pathways from avoidance habit to compulsive behavior severity outcomes across five domains: alcohol use (AUDIT), binge eating (BES),

binge watching (BWAQ), gambling (G-SAS), and obsessive-compulsive symptoms (OCI-R), while controlling for demographic factors (age, gender, years in education), apathy (AES), and still-valued cue responses.

Both depressive (BDI-II) (Table 3.3.a) and anxious (GAD-7) (Table 3.3.b) symptom severities independently mediated the relationship between avoidance habit and severity of compulsive behaviors in the same direction as the aggregated model. While effect sizes were marginally smaller in some cases, all patterns of significance were retained. These results support the validity of the aggregated negative emotionality approach and suggest that the severity of depressive and anxiety symptoms contributed comparably to the observed indirect effects.

| **Compulsivity Domain** | **Indirect Effect (b)** | **p-value** | **Confidence Intervals (CIs)** | **Model $R^2$** |
|---|---|---|---|---|
| Alcohol Use *(AUDIT)* | 0.05 | 0.02* | 0.01 to 0.08 | 0.11 |
| Binge Eating *(BES)* | 0.09 | 0.006** | 0.03 to 0.15 | 0.41 |
| Binge Watching *(BWAQ)* | 0.05 | 0.01* | 0.01 to 0.1 | 0.22 |
| Gambling *(G-SAS)* | 0.05 | 0.02* | 0.01 to 0.09 | 0.16 |
| Obsessive-Compulsive *(OCI-R)* | 0.1 | 0.006** | 0.03 to 0.17 | 0.39 |

**Table 3.3.a** Depressive Symptomatology (BDI-II) as Mediator

| **Compulsivity Domain** | **Indirect Effect (b)** | **p-value** | **Confidence Intervals (CIs)** | **Model $R^2$** |
|---|---|---|---|---|
| Alcohol Use *(AUDIT)* | 0.03 | 0.03* | 0.003 to 0.07 | 0.08 |
| Binge Eating *(BES)* | 0.07 | 0.01* | 0.02 to 0.12 | 0.31 |
| Binge Watching *(BWAQ)* | 0.05 | 0.02* | 0.008 to 0.09 | 0.2 |
| Gambling *(G-SAS)* | 0.05 | 0.02* | 0.007 to 0.09 | 0.17 |
| Obsessive-Compulsive *(OCI-R)* | 0.1 | 0.008** | 0.03 to 0.18 | 0.43 |

**Table 3.3.b** Anxiety Symptomatology (GAD-7) as Mediator

### *3.4: Assessing the Directionality of the Hypothesized Pathway via Reverse Mediation Analysis*

To test the directional specificity of the hypothesized pathway, we conducted a supplementary mediation analysis in which negative emotionality was specified as the predictor, habitual avoidance responding as the mediator, and compulsive symptom severity as the outcome. The

analysis controlled for demographic variables (age, gender, years of education), apathy (AES), and responses to still-valued cues post-devaluation (Table 3.4).

No significant indirect effects were observed in this reversed model. These findings support the directional assumption of the original mediation pathway, suggesting that habitual avoidance contributes to the emergence of negative emotional states, which in turn exacerbate compulsive symptoms—rather than habitual responding arising secondarily from elevated negative affect.

| **Compulsivity Domain** | **Indirect effect (b)** | **p-value** | **Confidence Intervals (CIs)** |
|---|---|---|---|
| Alcohol Use *(AUDIT)* | -0.007 | 0.54 | -0.03 to 0.02 |
| Binge Eating *(BES)* | 0.02 | 0.12 | -0.004 to 0.04 |
| Binge Watching *(BWAQ)* | 0.02 | 0.14 | -0.01 to 0.05 |
| Gambling *(G-SAS)* | -0.02 | 0.21 | -0.05 to 0.01 |
| Obsessive-Compulsive *(OCI-R)* | -0.01 | 0.27 | -0.03 to 0.01 |

**Table 3.4.** Assessing the Directionality of the Hypothesized Pathway via Reverse Mediation Analysis

*3.5: Specificity Analysis: Response Amounts to Still-Valued Stimuli*

To assess the specificity of the mediation model, we conducted a supplementary mediation analysis using avoidance responses to still-valued stimuli (i.e., stimuli still perceived as threatening) during the habit test as the predictor. Because these stimuli retained their aversive value, responses to them likely reflect goal-directed avoidance, in contrast to the habitual responding observed for devalued stimuli. If the mediation effects observed in the main model were driven by a general tendency to avoid threat, we would expect still-valued responses to similarly predict negative emotionality and compulsive behavior severity.

Avoidance of still-valued stimuli was not significantly associated with negative emotionality ($p = .83$) and did not contribute to any indirect effect on compulsive symptoms (Table 3.5). These findings suggest that the observed mediation effects are not simply a reflection of overall avoidance behavior or threat sensitivity. Rather, they point to a specific role for habitual avoidance—that is, persistent avoidance despite outcome devaluation—as a driver of elevated

negative emotionality, which in turn contributes to the severity of compulsive symptom expression.

| **Compulsivity Domain** | **Indirect effect (b)** | **p-value** | **Confidence Intervals (CIs)** |
|---|---|---|---|
| Alcohol Use *(AUDIT)* | -0.003 | 0.3 | -0.02 to 0.05 |
| Binge Eating *(BES)* | -0.006 | 0.29 | -0.03 to 0.1 |
| Binge Watching *(BWAQ)* | -0.004 | 0.3 | -0.02 to 0.06 |
| Gambling *(G-SAS)* | -0.003 | 0.3 | -0.02 to 0.05 |
| Obsessive-Compulsive *(OCI-R)* | -0.007 | 0.29 | -0.03 to 0.11 |

**Table 3.5.** Specificity Analysis: Response Amounts to Still-Valued Stimuli
